# Counting the non-existing: causes of death of undocumented migrants in Brussels-Capital Region (Belgium), 2005–2010

**DOI:** 10.1186/s13690-019-0369-6

**Published:** 2019-10-01

**Authors:** Dirk Lafaut, Hadewijch Vandenheede, Johan Surkyn, Gily Coene

**Affiliations:** 1 VUB, Pleinlaan 2, 1050 Brussels, Belgium; 2 VUB, Pleinlaan 5, 1050 Brussels, Belgium

**Keywords:** Cause of death, Cause-specific mortality risk, Undocumented migrants, Belgium, Health care access

## Abstract

**Background:**

So far knowledge about undocumented migrant health status is poor. The objective of this study is to compare patterns in causes of death between undocumented migrants and legal residents, of both migrant and non-migrant origin.

**Method:**

Using cause-of-death data, we compared undocumented migrants with Belgian residents and documented migrants through logistic regression analyses..

**Results:**

This study shows that male undocumented migrants have a significantly higher risk of death from cardiovascular diseases compared to male Belgian residents (OR: 1.37) and documented migrants (OR: 2.17). Male undocumented migrants also have an increased risk of dying from external causes of death compared to documented migrants (OR: 1.93). Furthermore, we found a lower risk of suicidal death in undocumented migrants compared to Belgian residents (OR men: 0.29, OR women: 0.15).

**Conclusions:**

We found important differences in underlying causes of death between undocumented migrants and residents in Belgium. These findings urge us to claim improved healthcare provision for undocumented migrants in Belgium.

**Trial registration:**

Medical ethics committee UZ Jette, Brussels, Belgium – Registration date: 18/05/2016 – Registration number: B.U.N. 143201628279.

## Background

Since 2005, research on health and access to care of undocumented migrants has been steadily increasing [34]. The main focus of this literature has been on the legal restrictions to healthcare access [4, 18, 29], on lower healthcare utilization [31] and on barriers that prevent the effective access to healthcare by undocumented migrants in Europe, Canada and the US [5, 10, 16]. These studies resulted in recommendations for policy changes to increase access to healthcare for this population and in proposing strategies to promote a better integration into mainstream healthcare institutions [29].

Yet, so far there is limited knowledge on the impact of an undocumented status on general health. There are no epidemiological data establishing the association between health policies and physical health outcomes of undocumented migrants [18]. Studies on chronic morbidity and especially mortality studies of undocumented migrants are rare. Most study designs are qualitative and much of the available research are reports, conducted by NGO’s. Because of their undocumented status, they are often not or not adequately registered in medical files, nor included in national databases [34] and they do not appear in international morbidity or mortality comparisons [13].

Reports such as those of the United Nations Monitoring Committee on Economic, Social and Cultural Rights describe the ill-health of undocumented migrants without referring to formal data (UN, 2000), information that is often reproduced in other publications [28]. Rousseau et al. [27] describe case reports of deaths that are considered to have been caused by problems in access to healthcare due to migration status concerns, as ‘wrongful deaths’. In addition, a lot of research in this area focuses on the illnesses of specific subgroups such as border crossers [33] or on specific pathologies and health issues. Especially, the effect of the migration status on mental health [16, 18, 30] and particularly vulnerable groups such as children and pregnant women have been studied. These studies explore prematurity, low birth weight and neonatal mortality [3, 9], work related accidents [16] or treatment of infectious diseases, such as hepatitis B/C, HIV and tuberculosis [8, 34]. Yet, focusing on specific pathologies does not provide us with insight into the impact of limited access to healthcare on overall health. Another approach is to measure the perceived health [5, 13] of undocumented migrants and use it as an indicator of health. These studies show that undocumented migrants more often report their health as poor or very poor compared to residents. Therefore, based on studies of Kaplan (1996) [12] and DeSalvo [6] that suggest a good general correlation between self-rated health and objective (and/or medical) indicators of health, they assume that morbidity and mortality among undocumented migrants is higher than among residents.

To sum up, information on morbidity and especially mortality of undocumented migrants, continues to be a major gap in knowledge about the health of undocumented migrants. This is puzzling, since one could either argue that their health and mortality is worse or better than that of legal residents, depending on which literature one draws upon. On the one hand, the hardships related with anti-immigration policies and the limited access to health services are perceived to result in worse health and higher mortality, especially from avoidable causes. On the other hand, according to the healthy migrant hypothesis, the all-cause and chronic-disease mortality of first-generation migrants is generally known to be lower than that of the host population [21, 25]. A good general health might be an alternative explanation for low utilisation rates of healthcare services. In order to inform policymakers adequately and to build up an inclusive healthcare system, the use of accurate health data is crucial.

Recently, Wahlberg et al. [32] were the first to determine national figures on causes of death of undocumented migrants, based on data of the Swedish causes of death register (CDR). They established that the mean age at death was much lower for undocumented migrants in Sweden compared to Swedish residents; and that they had an increased risk of dying from external causes and circulatory system diseases, and a lower risk of dying from neoplasms.

Belgium is well suited to study mortality by migrant background, as it has one of the largest proportions of immigrants in Europe [7]. The proportion of both documented and undocumented migrants in Belgium is largest in Brussels-Capital region [11, 23]. Belgium has a publicly financed healthcare system that covers all residents and documented migrants, whereas undocumented migrants can only access the public healthcare system after going through a complex parallel administrative procedure. Several federal and non-governmental reports state that there are many barriers for undocumented migrants to access healthcare in Belgium ([24]; Chauvin, 2009 [5]; Roberfroid et al. 2015 [26]), including the complexity of the administrative procedure, financial barriers and fear of arrest (Chauvin, 2009 [5]; Roberfroid et al. 2015 [26]).

The primary objective of this study is to establish whether the mortality pattern of undocumented migrants in the Brussels-Capital Region, when controlled for age and sex, differs from that of regular residents on the one hand and documented migrants on the other. Our study tests the findings of Wahlberg et al. [32]. Yet, our study also provides a more detailed analysis by performing age-adjustments (which were lacking in the work by Wahlberg) and by analysing a wider range of underlying causes of death. Furthermore, we compare patterns of causes of death in undocumented migrants not only with Belgian residents, but also with documented migrants.

## Methods

### How were the data gathered?

We performed a descriptive and multivariate analysis on cause-of-death data, stemming from two sources. One source is the Belgian death registration system which entails a statistical form filled out by a physician stating causes and circumstances of all deaths occurring on Belgian territory, regardless of the migration status of the deceased. Next, the Statistical Office establishes a linkage with the population register. Since death certificates are anonymous, linkage is based on common datafields in both databases. Death certificates that cannot be matched to a mortality event in the population register indicate the deceased was not known to that system and therefore did not belong to official population (‘unlinked group’). Based on nationality of birth, those that can be linked can further be divided into documented foreigners (‘documented group’) and residents of Belgian origin (‘residents group’). Death certificate data cover years 2005 to 2010.

The other source is the Belgian census of 2001. Again, only for the official population census and death certificates are linked, allowing cross-validation of the above-mentioned distinction between the linked and the unlinked group. We decided to focus on Brussels-Capital-Region as the available data didn’t allow us to generate national figures and by far the largest group of undocumented migrants in Belgium lives in the Brussels-Capital Region [19].

### Study population

From the unlinked group (*N* = 1051) we tried to extract those that most likely were undocumented migrants. In a first step we excluded those individuals who had Belgian nationality of birth, but had been deleted from the population register for administrative reasons (E.g. Belgian expats officially staying abroad who died during a visit in Belgium, longstanding loss of principal residence due to homelessness). In a second step we excluded individuals from countries of origin with no or very little visa-restrictions, most of whom were likely to be tourists, students or short term labour force. In addition, and similar to Wahlberg et al. [32], we excluded individuals from countries classified by WHO as group A (Table 1), which are countries with a low child and adult mortality [35]. The remaining individuals (*N* = 457) were considered as very likely to have been undocumented migrants (‘undocumented group’).

**Table 1:** Group A countries according to WHO classification of mortality stratum^a^

Andorra	Germany	Norway
Australia	Greece	Portugal
Austria	Iceland	San Marino
Belgium	Ireland	Singapore
Brunei Darussalam	Israel	Slovenia
Canada	Italy	Spain
Croatia	Japan	Sweden
Cuba	Luxembourg	Switzerland
Czech Republic	Malta	United Kingdom
Denmark	Monaco	USA
Finland	Netherlands	
France	New Zealand	

^a^WHO 2002

The documented migrant group consists of individuals, originating from the same group of countries as the undocumented group, yet with a link to the population register (*N* = 3450).

The residents group refers to all individuals with a Belgian nationality of birth who died in the Brussels Capital region in the period 2005–2010.

### Variables

We had information on the underlying cause of death, age at death, sex, and the exact date of death. Cases with missing information on these variables were removed from the analyses. Yet, there was little missing information (see Tables 2 and 3). Data about level of education were incomplete and hence, could not be used. The cause of death was coded according to the International Classification of Diseases 10th revision (ICD-10). Because of the relatively small numbers in the undocumented group we aggregated specific cancers (lung cancer, stomach cancer, pancreas cancer, ...) in an overarching group neoplasms (ICD-10 C00-D48). We took the same approach for infectious diseases (ICD-10 A00-B99) and cardiovascular diseases (ICD-10 I00-I99). We also aggregated the external causes of death. However, a sub analysis of the distinct external causes of death showed relevant findings, so we decided also to include specific data about suicide (ICD-10 X60-X84), homicide (ICD-10 X85-Y09), road accidents (ICD-10 V80-V89) and accidental falls (ICD-10 W00-W20) in the results-section, as they provide us with additional information about avoidable causes of death.

**Table 2:** Characteristics of study population of deceased undocumented migrants in the Brussels-Capital Region (period 2005–2010, Belgium)

	Men		Women		Total	
	n	%	n	%	n	%
Sex						
Determinable	307	67.2	150	32.8	457	100
Missing					1	0.01
Country of origin						
Morocco	54	19.7	20	14.0	74	17.8
Algeria	44	16.1	25	17.5	69	16.6
Congo	20	7.3	16	11.2	36	8.6
Other	156	56.9	82	57.3	238	57.1
Total	274	100.0	143	100.0	417	100.0
Missing	33	10.8	7	4.7	41	9.0
	n	Years	n	Years	n	Years
Age at death						
Mean	290	48.8	147	54.9	437	50.8
Std deviation		22.2		26.1		23.7
Missing					21

**Table 3:** Characteristics of study population of deceased documented migrants^a^ and Belgians in the Brussels-Capital Region (period 2005–2010)

Deceased documented migrants^a^							
		Men		Women		Total	
		n	%	n	%	n	%
Sex	Determinable	2051	59.5	1399	40.5	3450	100
Country of origin	Morocco	693	33.8	463	33.1	1156	33.5
	Algeria	46	2.2	24	1.7	70	2.0
	Congo	52	2.6	42	3.0	94	2.7
	Other	1260	61.4	870	62.2	2130	61.8
	Total	2051	100.0	1399	100.0	3450	100.0
		n	Years	n	Years	n	Years
Age at death	Mean	2051	66.9	1399	71.2	3450	68.6
	Std deviation		16.0		15.8		16.0
Deceased Belgians							
		Men		Women		Total	
		n	%	n	%	n	%
Sex	Determinable	21,440	44.2	27,021	55.8	48,511	100
		n	Years	n	Years	n	Years
Age at death	Mean	21,440	74.7	27,021	81.4	48,511	78.5
	Std deviation		14.2		12.9		13.9

^a^From same countries as deceased undocumented migrants

### Statistical analyses

We pooled the two datasets (linked and unlinked deaths), so we could compare mean age at death and the cause-of-death profile of the different groups. Since there were significant differences in mean age at death between the groups (cf. infra) we controlled the logistic regression analyses using cause-specific mortality as the dependent variable and undocumented vs documented/residents group as the independent variable for age at death. We stratified by sex, given the gendered patterns of mortality. Also, sensitivity analyses were performed to check the robustness of our results, especially in terms of controlling for the different age structure of the groups. Repeating analyses in more narrow age ranges (40–64 years or < 65 years) yielded similar results.

Our data do not provide information about duration of stay in Belgium of the deceased individuals in the documented and the undocumented group. Moreover, we have no information on the number and the age composition of the living population of undocumented migrants in the Brussels-Capital Region. Age composition is suspected to be much younger, partly explaining far lower mean ages at death in the undocumented group (cf. infra). Therefore, it was not possible to perform survival analyses.

### Ethical considerations

This research project was approved by the relevant medical ethics committee [BUN: 143201628279]. The data were anonymized in such a way that confidentiality was guaranteed. Data processing was done in accordance with the relevant Data Protection and Privacy legislation.

## Results

### Descriptive analysis of the study sample

From the unlinked death sample, 457 deaths were identified as presumably to have been those of undocumented migrants. About two thirds of them were males. The top 3 countries of origin were Morocco, Algeria and Congo (Tables 2 and 3).

As seen in Table 4, external causes were the most frequent underlying cause of death in undocumented men, amounting for 27.4% of all deaths. The second most common cause of death was cardiovascular diseases (26.4%), followed by neoplasms (23.5%). The most important underlying causes of death in undocumented women are neoplasms (37.3%) and cardiovascular diseases (22.7%). Compared to undocumented men, the relative frequency of external causes is much lower in undocumented women (8.7%). Similarly, among Belgian residents the frequency of external causes is far lower (men: 7.0%, women: 5.5%) compared to undocumented men. The same goes for documented migrants. The most important underlying causes of death in these groups are neoplasms and cardiovascular diseases.

**Table 4:** Frequency distribution of underlying causes of death among undocumented vs. documented migrants^a^ and Belgians in the Brussels-Capital Region (period 2005–2010)

Causes of death (ICD-10)	Men						Women					
	Undocumented		Documented		Belgian		Undocumented		Documented		Belgian	
	n	%	n	%	n	%	n	%	n	%	n	%
Infectious diseases	12	3.9	68	3.3	520	2.4	9	6.0	69	4.9	789	2.9
Neoplasms	72	23.5	522	25.5	6004	28.0	56	37.3	313	22.4	5977	22.1
Lung cancer	9	2.9	172	8.4	1651	7.7	8	5.3	34	2.4	856	3.2
Colon cancer	4	1.3	33	1.6	482	2.3	4	2.7	20	1.4	575	2.1
Breast cancer	/	/	/	/	/	/	10	6.7	59	4.2	1082	4.0
Prostate cancer	5	1.6	38	1.9	606	2.8	/	/	/	/	/	/
Cardiovascular diseases	81	26.4	437	21.3	6389	29.8	34	22.7	348	24.9	9053	33.4
External causes	84	27.4	179	8.7	1504	7.0	13	8.7	70	5.0	1476	5.5
Road accidents	10	3.3	32	1.6	121	0.6	2	1.3	6	0.4	64	0.2
Accidental falls	8	2.6	8	0.4	258	1.2	1	0.7	7	0.5	331	1.2
Suicide	10	3.3	36	1.8	470	2.2	3	2.0	15	1.1	258	1.0
Homicide	13	4.2	15	0.7	38	0.2	1	0.7	7	0.5	33	0.1

^a^From same countries as deceased undocumented migrants

### Undocumented migrants vs. Belgian residents

We compared the mean age at death and the cause-specific mortality of undocumented migrants and Belgian residents. The mean age at death (Tables 2 and 3) is lower in the undocumented group compared to residents. This is the case in both men and women (Men: undocumented: 48.8 years; residents: 74.7 - Women: undocumented 54.9 years; residents: 81.4).

Obviously, the lower mean age at death in undocumented migrants impacts their cause-of-death pattern. We therefore compared age-adjusted differences in patterns in causes of death stratified by sex (Table 5). Our findings show no increased risk of death from infectious diseases in undocumented migrants. Male undocumented migrants had a significantly higher risk of dying from cardiovascular diseases (OR: 1.37 (95% CI: 1.15–1.65)) compared to male Belgian residents. We also see a significantly lower risk of death from neoplasms (OR: 0.77 (95% CI: 0.65–0.95)) among male undocumented migrants. Overall, there was no higher risk of death from external causes of death amongst male undocumented migrants. There is a significantly reduced risk of suicide (OR: 0.29 (95% CI: 0.18–0.45)). The main finding amongst undocumented females is a significantly lower risk of death from external causes of death (OR: 0.62 (95% CI: 0.42–0.94)) and suicide (OR: 0.15 (95% CI: 0.07–0.35)) compared to female Belgian residents.

**Table 5:** Age-adjusted odds ratios for cause-specific mortality among undocumented migrants vs. Belgian residents in the Brussels-Capital Region (period 2005–2010)

Causes of death (ICD-10)	Odds ratios (OR)			
	Men		Women	
	OR^a^	95% CI	OR^a^	95% CI
Infectious diseases	1.38	0.87–2.18	1.27	0.74–2.21
Neoplasms	0.77	0.65–0.92	0.84	0.65–1.07
Cardiovascular diseases	**1.37**	**1.15–1.65**	1.16	0.89–1.50
External causes	1.09	0.87–1.36	0.62	0.42–0.94
Road accidents	0.74	0.43–1.29	0.72	0.27–1.91
Accidental falls	1.48	0.83–2.66	0.99	0.36–2.70
Suicide	0.29	0.18–0.45	0.15	0.07–0.35
Homicide	1.41	0.63–3.14	0.18	0.02–1.45

^a^*OR* Odds ratio

*p*<0.05

### Undocumented vs. documented migrants

Furthermore, we compared cause–specific mortality of undocumented migrants with that of documented migrants in the Brussels-Capital Region. The mean age at death was significantly lower in the undocumented group compared to the documented group (Men: undocumented 48.8 years; documented: 66.9 - Women: undocumented 54.9 years; documented: 71.2).

Our findings also show that male undocumented migrants had a significantly higher risk of dying from cardiovascular diseases (OR: 2.17 (95% CI: 1,60–2.95)), from external causes (OR: 1.93 (95% CI: 1.39–2.67)) and from accidental falls (OR: 5.50 (95% CI: 1.75–17.26)) compared to male documented migrants (Table 6). No differences in mortality from infectious diseases or neoplasms were found. Findings in women show a higher mortality from neoplasms in undocumented women versus documented women (OR: 1.51 (95% CI: 1.03–2.21)). Besides this, there are no statistically significant differences between documented and undocumented females.

**Table 6:** Age-adjusted odds ratios for cause-specific mortality among undocumented vs. documented migrants^a^ in the Brussels-Capital Region (period 2005–2010, Belgium)

Causes of death (ICD-10)	Odds ratios (OR)			
	Men		Women	
	OR^b^	95% CI	OR^b^	95% CI
Infectious diseases	1.19	0.61–2.32	0.89	0.42–1.94
Neoplasms	1.00	0.74–1.35	**1.51**	**1.03–2.21**
Cardiovascular diseases	**2.17**	**1.60–2.95**	1.32	0.86–2.01
External causes	**1.93**	**1.39–2.67**	0.47	0.21–1.06
Road accidents	1.09	0.74–1.57	0.82	0.12–5.87
Accidental falls	**5.50**	**1.75–17.26**	1.14	0.12–10.66
Suicide	0.55	0.24–1.27	0.26	0.05–1.44
Homicide	1.16	0.45–3.00	0.16	0.01–1.83

^a^From same countries as deceased undocumented migrants

^b^*OR* Odds ratio

*p*<0.05

## Discussion

About two thirds in the undocumented group were males, which is consistent with the composition of the sample in an earlier multi-country survey about the subjective health amongst undocumented migrants in Belgium [5]. Consistent with findings in the general population [1], our data reveal important sex differences in cause-specific mortality among undocumented migrants. The top 3 countries of origin in the undocumented group in our sample were Morocco, Algeria and Congo (Tables 1 and 2). Amongst the so-called third-countries, these nationalities are the most important countries of origin of undocumented migrants in Belgium [20].

For undocumented males, we found a significantly higher risk of dying from cardiovascular diseases compared with both Belgian residents and documented migrants. Deaths from heart diseases are considered to be avoidable, as they are both amenable and preventable. The increased mortality risk from cardiovascular diseases might be related to a reduced access to primary prevention or a reduced access to treatment for male undocumented migrants (Commission on Social Determinants of Health, 2008 [15];). Alternatively, the differences in cardiovascular mortality between male undocumented migrants and Belgian residents/documented migrants might partially be related to socio-economic factors, such as a difference in level of education between undocumented men and legal residents [14]. Unfortunately, our data did not provide sufficient data on socio-economic status to probe into this hypothesis; nor information on differences in lifestyle factors (such as smoking habits) that are likely to impact cardiovascular mortality.

When comparing mortality in male undocumented migrants to that of male Belgian residents we also found a decreased risk of death from neoplasms and suicide. However, this difference was not confirmed when comparing to documented migrants. Wahlberg et al. [32] also found a decreased risk of death from neoplasms for undocumented migrants compared to Swedish residents. Since mortality from neoplasms is comparable between documented and undocumented males, the decreased risk of death from neoplasms of undocumented migrants versus Belgian residents might point at a specific mortality profile of migrants -the healthy migrant effect- rather than to the undocumented status [21, 25].

Our findings also show a lower risk of suicidal death in both female and male undocumented migrants compared to Belgian residents. When comparing the risk of suicidal deaths with that of documented migrants, there were no statistically significant differences. These findings are remarkable, given the important body of literature suggesting an association between undocumented status and poor mental health outcomes such as depression, post-traumatic stress disorder and anxiety [16, 18, 30]. This literature is mainly based on qualitative research. Our findings rather seem to support the healthy migrant hypothesis, and more specifically ‘the migrant hope hypothesis’, when it comes to suicide risks of migrants. The latter hypothesis states that the hope that comes with migrating to a more ‘developed’ country engenders resilience towards otherwise intolerable conditions [2]. However, a lower suicide mortality risk doesn’t necessarily mean better mental health among undocumented migrants. The lower suicide mortality risk might also wear off with increasing duration of stay in Belgium. In any case, our findings point to the need to combine findings from quantitative and qualitative research to understand the mental health status of undocumented migrants better.

More than one quarter of the male undocumented migrants died from an external cause of death. A possible explanation for the increased risk of death from external causes and accidental falls among undocumented men versus documented migrants, is that working conditions of male undocumented migrants are worse than those of documented migrants who are likely to have a higher socioeconomic status. Undocumented migrant men in Brussels play an important role in the informal economy, mainly in construction. Safety issues in this sector have repeatedly been reported [22]. Undocumented women mainly work in childcare and domestic work, which could explain the gender difference [36]. Contrary to Wahlberg et al. [32], who found an increased risk of death from external causes among both male and female undocumented migrants, we surprisingly found a lower risk of death from external causes amongst female undocumented migrants, compared to Belgian women.

In contrast to male undocumented migrants we do not find statistically significant differences between female undocumented migrants and Belgian women/documented migrants for most underlying causes of death. There are several possible explanations for these different findings in undocumented males and females. First, the sample size of the female undocumented migrants was relatively small. Consistent with the higher number of male undocumented migrants in Belgium [5], the male sample was twice as large as the female sample. Another possible explanation for the important gender differences is the different access to healthcare and shelters, which has been described in qualitative research. Despite equal health rights, undocumented women seem to have better access to healthcare and social care through informal practices identifying women as victims (Author, forthcoming). Possibly, the differences also reflect a difference in the population characteristics or differences in living and working conditions between undocumented males and females. These different factors could (partially) account for the gendered differences in mortality risks. Similarly, earlier research shows that migration-related health inequalities can affect man and women differently [17].

### Strengths and limitations

Several literature reviews mention the lack of reliable epidemiological data about health outcomes of undocumented migrants [18, 34]. To our knowledge, this is one of the first studies providing information about underlying causes of death among (Belgian) undocumented migrants. Wahlberg et al. [32] did similar research on Swedish undocumented migrants, however they could not account for age, which is essential given the effect age structure has on cause of death patterns. We also performed analyses for a wider range of underlying causes of death, and compared data about undocumented migrants with both residents and documented migrants.

Nevertheless, this study has several limitations. Firstly, we have no information on the population of undocumented migrants. Therefore we could not estimate mortality rates. Secondly methodologically, identifying the undocumented migrants in the ‘unlinked group’ could induce bias. However, since most WHO group A countries do not have visa restrictions it is unlikely that we excluded undocumented migrants. Moreover, the composition of the sample was similar to earlier surveys about perceived health among undocumented migrants in Belgium. A third limitation is that the numbers in the undocumented group are relatively small. As a consequence, the comparison of the causes of death in aggregated groups provides little detail about what specific illnesses contribute to the difference. Fourthly, data about level of education and socio-economic status could not be corrected for as these were too limited. Lastly, some of the differences in causes of death between the groups could be explained by the difference in mean age at death. We performed age-adjustments, but this technique has limitations, especially when comparing groups with very different age composition. However, even if we restricted the analyses to people under 65 years of age or limited the age range from 40 to 64, relative differences in causes of death between the groups remained intact, which is showing the robustness of the results.

## Conclusions

This study shows important differences in the mean age at death and underlying causes of death in undocumented migrants compared to Belgian residents and documented migrants.

Most importantly, male undocumented migrants in Belgium have a statistically significant increased risk of dying from cardiovascular diseases. Our study does not allow to determine whether this is caused by lifestyle factors, reduced access to primary prevention, reduced access to treatment, or a combination of these. This requires further research.

Nevertheless, our analyses on causes of death provide information on lacunas in health services. Cardiovascular deaths are considered a source of avoidable mortality. This excess risk of cardiovascular death could be avoided through better access to healthcare and improving wider determinants of health such as lifestyle factors, daily living conditions and socio-economic status. These findings indicate that, to address the issue of poor health among undocumented migrants, we must rather improve general healthcare provision for undocumented migrants and focus on social determinants of health, rather than focussing on improving health services for specific illnesses or particularly ‘vulnerable’ groups.

## Data Availability

The data that support the findings of this study are available from Interface Demography – VUB, but restrictions apply to the availability of these data, which were used under license for the current study, and so are not publicly available. Data are however available from the authors upon reasonable request and with permission of Interface Demography – VUB.

## Abbreviations

BUN: Belgisch uniek nummer; [English: Unique Number of registration in Belgian medical ethics committee]
CDR: Causes of Death Register
CI: Confidence Interval
CSDH: *Commission on Social Determinants of Health*
FRA: European Union Agency for Fundamental Rights
ICD: International Classification of Diseases
KCE: Belgian Health Care Knowledge Centre
MdM: Médecins du Monde
OR: Odds Ratio
ORCA: Organisatie voor Clandestiene Arbeidsmigranten [English: Belgian NGO on fair work]
PICUM: *Platform for International Cooperation on Undocumented Migrants*
POD MI: Programmatorische Overheidsdienst Maatschappelijke integratie; [English: *Federal* Public Service for Social Integration]
UN: United Nations
WHO: World Health Organization
